# Determinants associated with low dietary diversity among migrants to Morocco: a cross sectional study

**DOI:** 10.1038/s41598-024-59082-8

**Published:** 2024-04-10

**Authors:** Firdaous Essayagh, Meriem Essayagh, Abdellah Lambaki, Ahmed Anouar Naji, Sanah Essayagh, Touria Essayagh

**Affiliations:** 1 Université Sidi Mohamed Ben Abdellah, Laboratoire droit privé et enjeux de développement, Faculté des sciences juridiques, économiques et sociales, Fès, Morocco; 2 Office national de sécurité sanitaire des produits alimentaires, Oriental, Morocco; 3 Université de Lomé, Faculté des sciences de la santé, Lomé, Togo; 4 Hassan First University of Settat, Faculté des sciences et techniques, Laboratoire agroalimentaire et santé, Settat, Morocco; 5 Hassan First University of Settat, Institut supérieur des sciences de la santé, Laboratoire sciences et technologies de la santé, Settat, Morocco

**Keywords:** Health occupations, Medical research, Risk factors

## Abstract

Low dietary diversity (LDD) is prevalent among vulnerable populations, posing a morbidity risk. Few studies have been conducted on the dietary diversity of migrants. The objectives of this study are to determine the prevalence of LDD among migrants in Morocco and the risk factors associated with it. In the Oriental region, we conducted a cross-sectional study with migrants between November and December 2021. The sampling method used was convenience sampling. A face-to-face, structured questionnaire was used to collect sociodemographic, behavioral, and clinical data. We calculated a dietary diversity score based on a 24-h food recall and assessed food intake. The risk factors associated with LDD were identified using multivariate logistic regression. A total of 445 migrants was enrolled. The prevalence of LDD was 31.7%. Risk factors associated with LDD were: being homeless (adjusted Odds Ratio (AOR) of 6.32; CI% [3.55–11.25]), a lack of social support (AOR of 2.30; CI% [1.33–03.98]), and low monthly income (AOR of 8.21; CI% [3.39–19.85]). Public policies must focus on social and environmental determinants. Nutrition training programs should be set up for the migrant population.

## Introduction

Ending hunger and achieving food security and adequate nutrition are part of the Sustainable Development Goals^1^. Access to adequate, safe, nutritious, and diverse food is a basic human right for everyone, regardless of gender, race, religion, or political beliefs^2^. Wars, conflicts, intra-community violence, and repression continue to undermine the ability of people to maintain their day-to-day livelihoods and generate large numbers of asylum seekers and refugees. In 2020, worldwide, 281 million international migrants were registered, including four million asylum seekers and 33.8 million refugees^3,4^. Human mobility aggravates the food and nutritional vulnerability and leads to food insecurity. In 2019, two billion people worldwide lacked regular access to safe, nutritious, and sufficient food^5^.

Vulnerable populations such as migrants may be exposed to insufficient dietary diversity and inadequate nutrition. However, the literature reports that a diversified diet would improve survival, prevent malnutrition and reduce diseases such as diabetes, depression, asthma, metabolic syndrome and osteoporosis^6^. This dietary diversity determines diet quality and nutritional adequacy because no single food can meet an individual's nutritional needs^7^.

Studies on the migrant population in Morocco have shown various nutrition-related issues. Overweight and obesity are prevalent in 39.2% of the population^8^, as are respiratory tract infections (13%) and gastrointestinal illnesses (9%)^9^.

Since implementing a new migrant policy characterized by respect for the human rights of people on the move in 2013, Morocco became not only a transit country to Europe but also a host country for people from Syria and West and Central Africa, with 86,000 migrants in 2014^4^. To date, research has identified the determinants of dietary diversity among adolescents, pregnant and lactating women, and children under the age of five, but no epidemiological study in Morocco has investigated dietary diversity among undocumented migrants, asylum seekers, and refugees^10–13^.

Taking an interest in dietary diversity among the migrant population would make it possible to maintain or even improve their state of health, which would have a long-term positive influence on the socio-economic capital of the host country. Hence, the objective of our study is to measure the prevalence of low dietary diversity among migrants in the Oriental region of Morocco and to identify the associated risk factors.

## Methods

### Setting

The Oriental Region in Morocco has an area of 90,130 km^2^ and has a population of 2,314,346 habitants^14^. It is limited to the South-East by Algeria and to the North by the Mediterranean Sea and the countries of Southern Europe. The region represents a gateway to Europe^15^. It is one of the three main regions hosting asylum seekers and refugees, along with the Rabat-Salé-Kénitra region and the Tangier Tétouan El Houcima region^16^.

### Study design

We conducted a cross-sectional survey in the Oriental region between November and December 2021. We used convenience sampling. A first draw was carried out to select 17 organizations among 30 organizations working in favor of migrants and people in vulnerable situations in the Oriental region. A second draw was carried out among migrants aged 18 and over, present on the day of the survey in the selected organizations, and who had given their written consent to participate in the survey.

### Population and sample size

In this study, a migrant was defined as any person of foreign origin, without legal status in Morocco, regardless of their date of entry into the country and the duration of their stay, or even settlement. Migrants were classified into three categories: (i) Undocumented migrants i.e. those who do not have a valid Moroccan residence permit; (ii) Asylum seekers, defined as anyone seeking safety from persecution or harm in a country other than their own and awaiting a response on their application for refugee status; and (iii) Refugees, defined as anyone who is recognized by the host country as unable or unwilling to return to their country of origin due to a well-founded fear of persecution on account of their race, religion, nationality, membership in a social group, or political opinions^4^.

The minimum size of our sample was estimated at 384 migrants. For the calculation of the sample size, we used the following formula: ${\text{n}} = \frac{{z^{2} p\left( {1 - p} \right)}}{{e^{2} }}$, with an estimated prevalence of low dietary diversity (p) of 50%, a confidence interval (z) of 95%, and a margin of error (e) of 5% (Epi-Info version 7).

### Data source

The trained investigators discussed the objective of the study with potential participants who were present in the selected organizations during the days of the survey and invited them to participate in it. Following a brief conversation with potential participants, those who satisfied the inclusion criteria and gave their agreement were invited to a face-to-face interview. Data collection took place in a closed room in the organization to ensure confidentiality and anonymity. The survey was done throughout the week, including Saturdays and Sundays, to reduce healthy workers bias.

A structured, standardized anonymous questionnaire was administered by investigators during a face-to-face interview with the participant to collect data in relation to socio-economic, demographic, behavioral, and clinical characteristics. The questionnaire was administered in Arabic, French, or English which corresponds to the main languages spoken by migrants in Morocco. Investigators were trained in data collection to limit measurement bias. No remuneration was awarded to participants to limit selection bias.

### Variables

The socio-economic and demographic characteristics collected were: sex (female or male), age in years, marital status (being single or having partnered), educational level (illiterate, elementary, middle school, high school, and college), native country (Eastern Mediterranean Region, Sub-Saharan Africa), housing type (homeless or apartment), number of persons per house (≥ 10, between five and nine and more than five), occupation (no, yes), monthly income ($) (≤ 150 or more than 150), health insurance (no or yes), length of stay in Morocco in years (< 5 or ≥ 5), legal status (undocumented migrant, asylum seeker, and refugee), and number of countries crossed (≥ 3 or < 3).

Dietary diversity score is a qualitative measure of food consumption that reflects the nutritional adequacy of the diet for the individual. It describes how much a person consumes diverse groups of food^17^. This study adopted the Food and Agriculture Organization of the United Nations Dietary Diversity Score Measurement Questionnaire^17^. Food data was collected using the recall method based on a list of twenty-four categories of foods eaten in the last 24 hours^17^.These foods have been grouped into nine groups: (1) cereals; (2) white roots and tubers; (3) vegetables (including vitamin A-rich tubers, dark green leafy vegetables, and other vegetables); (4) fruits; (6) eggs; (7) fish and seafood; (8) legumes, nuts, and seeds; and (9) milk and milk products. For mixed dish consumption, we asked participants to name the list of ingredients included in the dish, and we recorded them separately in the food list. If one or more food groups were not mentioned by the participants after the recall, they were asked if they had consumed one or more foods from these groups in case they had forgotten. We noted the value of "1" when the food of a group was consumed and the value of "0" otherwise. Dietary diversity scores were calculated by counting the number of food groups participants consumed over a 24-h period. The individual dietary diversity score ranged from 0 to 9. As there are no established limits indicating the number of food groups at which dietary diversity is considered adequate or inadequate, we have based our consensus on the value of four. A participant was classified as having low dietary diversity when they consumed four or fewer food groups from the nine food groups in the past 24 h. Above four, the participant was classified as having adequate dietary diversity.

Symptoms of anxiety and depression were assessed using the Hospital Anxiety and Depression scale. On this scale, the participant stated how he or she felt over the past two weeks. The Anxiety sub-scale consists of seven items: (i) I am tense or irritable; (ii) I am afraid that something bad will happen to me; (iii) I worry; (iv) I can sit quietly doing nothing and feel relaxed; (v) I am afraid and my stomach is knotted; (vi) I am on the move and can't stop; and (vii) I have sudden feelings of panic. Each of these items was given a score ranging from 0 to 3, with "0" signifying no symptoms and "3" indicating the most severe symptoms. The sub-scale for anxiety varied from 0 to 21. A score of 11 or above was considered to indicate elevated levels of anxiety symptoms^18^.

The Depression sub-scale consists of seven items: This sub-scale includes seven items: (i) I continue to enjoy the same things I used to; (ii) I laugh easily and see the bright side of things; (iii) I'm in a good mood; (iv) I feel like I'm idle; (v) I'm no longer bothered by how I look; and (vi) I look forward to doing certain activities. (vii) I appreciate both good books and good radio or television shows. Each of these items received a score ranging from 0 to 3. The sub-score for total depression varied from 0 to 21, with a "0" indicating no symptoms and a "3" indicating the most severe symptoms. A score of 11 or above was considered to indicate elevated levels of depression symptoms^18^.

Physical activity was measured according to Global Physical Activity Questionnaire (GPAQ)^19^. Physical activity deemed unsatisfactory if it occurred less than five times per week and for less than 30 min per day^19–22^. Social support was notified when the participant reported receiving support from close friends or organizations^23^.

### Statistical methods

Epi Info version 7.2.0.1 was used to enter and analyze data. All tests were two-sided and statistical significance was set at a *p*-value less than 0.05. During the descriptive analysis, the categorical variables were expressed in number and percentage, and the continuous variables were expressed in mean and standard deviation. The proportions of categorical variables were compared using the chi-square test or Fisher's exact test, where applicable. Continuous variables were compared using the analysis of variance test. We included in the multiple logistic regression any variables with a *p*-value < 0.05 in the bivariate analysis. Multivariate logistic regression was used to identify risk factors associated with low dietary diversity, providing odds ratios (ORs) and adjusted ORs with 95% Confidence Intervals (CIs).

### Ethics approval and informed consent to participate

The study adhered to the Helsinki Declaration. Potential participants were informed of the objectives of study and procedure. All subjects who took part in the study signed an informed written statement of consent. The study protocol was reviewed and approved by the ethical review board of the faculty of medicine and pharmacy in Rabat, Morocco (#34/21).

## Results

### Socio-economic and demographic characteristics

The study included 445 migrants, with 141 (31.7%) having low dietary diversity. The participants' average age was 27.9 ± 10.9 years, ranging from 18 to 73 years. A total of 306 (68.8%) were males, and 139 (31.2%) were females. There were 228 undocumented migrants (51.2%), 305 single migrants (68.5%), 112 illiterates (25.2%), and 109 homeless (24.5%). A total of 221 (49.7%) participants had no social support, and 300 (67.4%) had a monthly income of less than or equal to $150 (Table 1).

**Table 1:** Socio-economic and demographic characteristics among migrants, Morocco, 2021.

Variables	All participants n (%)	Low dietary diversity n (%)	Adequate dietary diversity n (%)	*p-value*
All participants	445 (100)	141 (31.7)	304 (68.3)	
Age group in years				<0.001
18–20	119 (26.7)	65 (46.1)	54 (17.8)	
21–25	120 (27.0)	40 (28.4)	80 (26.3)	
26–30	85 (19.1)	12 (08.5)	73 (24.0)	
≥31	121 (27.2)	24 (17.0)	97 (31.9)	
Sex				<0.001
Male	306 (68.8)	118 (83.7)	188 (61.8)	
Female	139 (31.2)	23 (16.3)	116 (38.2)	
Marital status				0.006
Being single^‡^	305 (68.5)	109 (77.3)	196 (64.5)	
Partnered^†^	140 (31.5)	32 (22.7)	108 (35.5)	
Education				<0.001
Illiterate	112 (25.2)	22 (15.6)	90 (29.6)	
Elementary	144 (32.4)	59 (41.9)	85 (27.9)	
Middle school	56 (12.6)	26 (18.4)	30 (09.9)	
High school	92 (20.6)	24 (17.0)	68 (22.4)	
College	41 (09.2)	10 (07.1)	31 (10.2)	
Native country				0.03
Eastern mediterranean region	100 (22.5)	23 (16.3)	77 (25.3)	
Sub-Saharan Africa	345 (77.5)	118 (83.7)	227 (74.7)	
Length of stay in Morocco in years				<0.001
<5	351 (78.9)	128 (90.8)	223 (73.4)	
≥5	94 (21.1)	13 (09.2)	81 (26.6)	
Legal status				<0.001
Undocumented migrant	228 (51.2)	98 (69.5)	130 (42.8)	
Asylum seeker	177 (39.8)	36 (25.5)	141 (46.4)	
Refugee	40 (09.0)	07 (05.0)	33 (10.8)	
Number of countries crossed (n=430)				0.15
≥3	134 (31.2)	36 (26.5)	98 (33.3)	
<3	296 (68.8)	100 (73.5)	196 (66.7)	
Housing type				<0.001
Homeless	109 (24.5)	83 (58.9)	26 (08.6)	
House*	336 (75.5)	58 (41.1)	278 (91.4)	
Number of persons per house (n=336)				0.55
≥10	40 (11.9)	6 (10.4)	34 (12.2)	
5–9	174 (51.8)	34 (58.6)	140 (50.4)	
<5	122 (36.3)	18 (31.0)	104 (37.4)	
Occupation				
No	430 (96.6)	138 (97.9)	292 (96.0)	0.32
Yes	15 (03.4)	3 (02.1)	12 (04.0)	
Monthly income ($)				<0.001
≤150	300 (67.4)	135 (95.7)	165 (54.3)	
>150	145 (32.6)	6 (04.3)	139 (45.7)	
Health insurance				0.14
No	444 (99.8)	140 (99.3)	304 (100.0)	
Yes	1 (00.2)	1 (00.7)		
Social support				<0.001
No	221 (49.7)	109 (77.3)	112 (36.8)	
Yes	224 (50.3)	32 (22.7)	192 (63.2)	

*sd* standard deviation.

^†^A partnered means to be married or to be in concubine.

^‡^Being single means to be single or divorced or widower.

*House means living in house or apartment or reception center. For categorical variables, the Pearson chi-2 test estimated the association between the depending variable and the independent variables when the conditions were valid. For the continuous variables, we used a comparison test of two means; *p*-value was considered significant when it was less than 0.05.

### Meal frequency and clinical characteristics

Table 2 summarizes meal frequency and clinical characteristics among migrants. A total of 211 (47.4%) participants consumed at most two meals per day, 178 (40.0%) had symptoms of depression, 44 (9.9%) had unsatisfactory physical activity, and 177 (39.8%) were overweight or obese.

**Table 2:** Meal frequency and clinical features of the migrants, Morocco, 2021.

Variables	All participant (n = 445)	Low dietary diversity n (%) (n = 141)	Adequate dietary diversity n (%) (n = 304)	*p-value*
Number of meals consumed per day				< 0.001
≤ 2	211 (47.4)	106 (75.2)	105 (34.5)	
> 2	234 (52.6)	35 (24.8)	199 (65.5)	
Symptoms of anxiety				0.013
Yes	174 (39.1)	67 (47.5)	107 (35.2)	
No	271 (60.9)	74 (52.5)	197 (64.8)	
Symptoms of depression				< 0.001
Yes	178 (40.0)	78 (55.3)	100 (32.9)	
No	267 (60.0)	63 (44.7)	204 (67.1)	
Physical activity				0.09
Unsatisfactory	44 (09.9)	9 (06.4)	35 (79.5)	
Satisfactory	401 (90.1)	132 (93.6)	269 (88.5)	
Overweight/obesity				< 0.001
Yes	177 (39.8)	34 (24.1)	143 (47.0)	
No	268 (60.2)	107 (75.9)	161 (53.0)	
Diabetes				0.23
Yes	18 (04.0)	8 (05.7)	10 (03.3)	
No	427 (96.0)	133 (94.3)	294 (96.7)	
Hypertension				0.60
Yes	124 (27.9)	37 (26.2)	87 (28.6)	
No	321 (72.1)	104 (73.8)	217 (71.4)	

For categorical variables, the Pearson chi-2 test estimated the association between the depending variable and the independent variables when the conditions were valid; *p*-value was considered significant when it was less than 0.05.

### Food groups

The distribution of the consumption of the food groups most frequently consumed by migrants showed that 433 (97.3%) of the migrants consumed cereals, 401 (90.1%) spices, condiments, and drinks, 366 (82.2%) vegetables, and 361 (81.1%) milk and dairy products (Table 3).

**Table 3:** Consumption of food groups among migrants, Morocco, 2021.

Variables	All participants n (%) (n = 445)	Low dietary diversity n (%) (n = 141)	Adequate dietary diversity n (%) (n = 304)
Cereals			
Yes	433 (97.3)	130 (92.2)	303 (99.7)
No	12 (02.7)	11 (07.8)	1 (00.3)
White roots and tubers			
Yes	189 (42.5)	21 (14.9)	168 (55.3)
No	256 (57.5)	120 (85.1)	136 (44.7)
Vegetables			
Yes	366 (82.2)	74 (52.5)	292 (96.0)
No	79 (17.8)	67 (47.5)	12 (04.0)
Fruits			
Yes	274 (61.6)	40 (28.4)	234 (77.0)
No	171 (38.4)	101 (71.6)	70 (23.0)
Meats			
Yes	243 (54.6)	19 (13.5)	224 (73.7)
No	202 (45.4)	122 (86.5)	80 (26.3)
Eggs			
Yes	309 (69.4)	52 (36.9)	257 (84.5)
No	136 (30.6)	89 (63.1)	47 (15.5)
Fish and seafood			
Yes	61 (13.7)	3 (02.1)	58 (19.1)
No	384 (86.3)	138 (97.9)	246 (80.9)
Legumes, nuts and seeds			
Yes	45 (10.1)	1 (00.7)	44 (14.5)
No	400 (89.9)	140 (99.1)	260 (85.5)
Milk and dairy products			
Yes	361 (81.1)	101 (71.6)	260 (85.5)
No	84 (18.9)	40 (28.4)	44 (14.5)

### Dietary diversity

The overall prevalence of low dietary diversity (LDD) among migrants was 31.7%, while the overall prevalence of adequate dietary diversity was 68.3%.

The cut-off *p*-value after the bivariate analysis was set at *p*-value < 0.05. According to the bivariate analysis, the following factors were associated with low dietary diversity: (i) age (*p* < 0.001), (ii) male sex (*p* < 0.001); (iii) being single (*p* = 0.006); (iv) low level of education (*p* < 0.001), (v) native of countries in the eastern Mediterranean region (*p* = 0.03); (vi) length of stay in Morocco less than five years (*p* < 0.001), (vii) undocumented migrants (< 0.001); (viii) homeless (*p* < 0.001), (ix) monthly income below $150 (*p* < 0.001); (x) lack of social support (*p* < 0.001); (xi) consumption of no more than two meals per day (*p* < 0.001); (xii) the symptoms of anxiety (*p* = 0.013); and (xiii) the symptoms of depression (< 0.001); and (ivx) overweigh/obesity (< 0.001) (Table 4).

**Table 4:** Multivariate analysis (odds ratio, *p*-value) of risk factors of low dietary diversity among migrants, Morocco, 2021.

Variables	Bivariate analysis		Multivariate analysis complete model		Multivariate analysis final model	
	COR [95% CI]	*p-*value	AOR [95% CI]	*p-*value	AOR [95% CI]	*p-*value
Age in years		< 0.001			********	***
18–20 ≥ 31	4.86 [2.73–8.64]	< 0.001	0.93 [0.34–2.54]	0.89	********	***
21–25 ≥ 31	2.02 [1.12–3.63]	0.01	1.11 [0.45–2.72]	0.80	********	***
26–30 ≥ 31	0.66 [0.31–1.41]	0.28	0.35 [0.13–0.96]	0.04	********	***
Sex male/female	3.16 [1.91–5.23]	< 0.001	0.82 [0.40–1.67]	0.59	********	***
Marital status being single/partnered	1.87 [1.18–2.96]	0.006	0.55 [0.24–1.24]	0.15	********	***
Education		< 0.001			********	***
Illiterate/college	0.75 [0.32–1.77]	0.52	0.91 [0.27–3.06]	0.88	********	***
Elementary/college	2.15 [0.98–4.72]	0.05	1.03 [0.33–3.24]	0.94	********	***
Middle school/college	2.68 [1.10–6.51]	0.02	1.11 [0.33–3.75]	0.85	********	***
High school/college	1.09 [0.46–2.56]	0.83	1.06 [0.31–3.57]	0.91	********	***
Native of Eastern Mediterranean region/Sub-Saharan region	0.57 [0.34–0.96]	0.03	1.12 [0.43–2.91]	0.80	********	***
Length of stay in Morocco < 5 years/ ≥ 5 years	3.57 [1.91–6.67]	< 0.001	1.64 [0.72–3.71]	0.23	********	***
Legal status		< 0.001			********	***
Undocumented migrant/refugee	3.55 [1.50–8.37]	0.003	1.84 [0.55–6.16]	0.31	********	***
Asylum seeker/refugee	1.20 [0.49–2.94]	0.68	1.72 [0.50–5.95]	0.38	********	***
Homeless/house	15.3 [9.06–25.8]	< 0.001	4.48 [1.99–10.0]	0.0003	6.32 [3.55–11.25]	< 0.001
Monthly income less than or equal to 150 ($)/ > 150 ($)	18.9 [8.10–44.2]	< 0.001	7.43 [2.92–18.8]	< 0.001	8.21 [3.39–19.85]	< 0.001
Lack of social support	5.83 [3.69–9.22]	< 0.001	2.39 [1.25–4.56]	0.008	2.30 [1.33–03.98]	0.0026
Number of meals consumed per day ≤ 2/ > 2 per day	5.73 [3.66–8.99]	< 0.001	2.30 [1.22–4.34	0.01	********	***
Presence of anxiety symptoms /absence of anxiety symptoms	1.66 [1.11–2.50]	0.013	1.75 [0.98–3.14]	0.06	********	***
Presence of depression symptoms /absence of depression symptoms	2.52 [1.67–3.80]	< 0.001	1.09 [0.62–1.92]	0.75	********	***
Presence of overweigh or obesity/ Absence of overweigh or obesity	0.35 [0.22–0.55]	< 0.001	0.83 [0.44–1.57]	0.57	********	***

*COR* Crude Odds Ratio; *AOR* Adjusted Odds Ratio; *CI* Confidence Interval. The denominator is the reference group.

### Multivariate analysis

After adjusting for the other variables, three variables were identified as risk factors associated with low dietary diversity: (i) homeless were 6.32 (95% CI [3.55–11.25]) more likely to have low dietary diversity than migrants with house; (ii) migrants without social support were 2.30 (95% CI [1.33–03.98]) more likely to have low dietary diversity than migrants who have social support ; and (iii) migrants with monthly income ≤ 150 ($) were 8.21 (95% CI [3.39–19.85]) more likely to have low dietary diversity than migrants with monthly income > 150 ($) (Table 4).

## Discussion

To our knowledge, this survey is the first in Morocco to measure the prevalence of low dietary diversity among undocumented migrants, asylum seekers, and refugees. This prevalence is 31.7%. When we compared the prevalence of our study to that of Algeria, a neighboring country with the same socio-economic characteristics as Morocco, we found that in Algeria, in 2014, for 355 migrants, the prevalence of low dietary diversity was 60.0%^24^.

In our study, being homeless was a risk factor associated with low dietary diversity. This could be explained by the unfavorable environmental conditions marked by the absence of an adequate place for the preparation of food, the lack of hygiene measures, and the lack of storage spaces, which would limit the preparation of meals and lead to low dietary diversity. The lack of social support could lead migrants to obtain cheaper food, and lower quality food, and have a monotonous and undiversified diet. In our study, a lack of social support was associated with low dietary diversity.

In the current work, the low income of the migrant population was associated with low dietary diversity. The same finding was reported in a study from Kenya in 2018^25^. Indeed, the global economic and financial crisis, poor buying power, and rising food costs would encourage individuals to avoid purchasing particular food products^26^. Poverty would also be at the origin of the adoption of a monotonous diet, of the adoption of a strategy based on staggering meal times and consuming little food, and the consumption of processed foods rich in saturated fats, salt, and sugar. Vulnerable populations, including migrants, would turn to less diversified, less expensive foods, which certainly fill the stomach but are less nutritious, which weakens their immune systems, makes them more exposed to diseases, and reduces their productivity^26^.

Ghattas et al. report in a study of 639 Iraqi migrants a low consumption of milk and dairy products, fruits, meat, and fish, and a high dependence on cereals and fats^27^. These results are consistent with the results of our study. This could be explained by the fact that milk and dairy products, fruits, meat, and fish have relatively high prices compared to cereals, and oils and are therefore the first foods to be reduced in case of financial difficulties encountered by migrants in host countries. It might also possibly be because these items require cool storage to prevent deterioration, which is unlikely to be available to homeless people or those on limited budgets.

### Limitations

Our study had certain limitations, notably its cross-sectional design. Certainly, it is quick, simple, and low-cost; nevertheless, it presents difficulties in confirming the causal association between exposure and low dietary diversity when assessed simultaneously.

Other limitations were noted: the food variety score was obtained qualitatively, without quantification of foods consumed in each food group; the social desirability bias in response to monthly income and behavioral characteristics was also reflected during data collection, despite the promise of anonymity and the use of community actors; and the scarcity of studies relating to dietary diversity among the migrant population has made discussion difficult.

## Conclusion

In the literature, there is a dearth of epidemiological studies on diet among migrants. Our study was able to meet its objectives, which were to determine the prevalence of low dietary diversity among migrants and the risk factors associated with it.

Undocumented migrants, asylum seekers, and refugees are a vulnerable group with limited dietary options. Low dietary diversity was associated with risk factors such as homelessness, lack of social support, and low monthly income.

Following these findings, nutrition training programs should be developed and implemented to improve migrants' nutritional and dietary knowledge and to broaden the variety of foods and food groups available to ensure nutritional adequacy. Nutrition training might be a good strategy if the migrant population's financial and living conditions improve at the same time. Similar studies should be conducted in various regions of Morocco to ensure that the findings are generalizable.

## Data Availability

All data generated or analyzed during this study are included in this published article.
